# Levels of circulating TNF-related apoptosis-inducing ligand in celiac disease

**DOI:** 10.3892/etm.2014.2026

**Published:** 2014-10-15

**Authors:** CLAUDIO CELEGHINI, TARCISIO NOT, ALESSIA NORCIO, LORENZO MONASTA, PAOLA SECCHIERO

**Affiliations:** 1 Department of Life Sciences, University of Trieste, Trieste I-34138, Italy; 2 Institute for Maternal and Child Health - IRCCS ‘Burlo Garofolo’, Trieste I-34137, Italy; 3 Department of Morphology, Surgery and Experimental Medicine and LTTA Centre, University of Ferrara, Ferrara I-44121, Italy

**Keywords:** TNF-related apoptosis-inducing ligand, celiac disease, gluten-free diet, type 1 diabetes mellitus

## Abstract

It has previously been demonstrated that the circulating levels of TNF-related apoptosis-inducing ligand (TRAIL) are significantly lower in patients with type 1 diabetes (T1D) than in normal age- and gender-matched controls. Since celiac disease (CD) is often associated with T1D, a retrospective study was performed to analyze the sera of a cohort of pediatric subjects: i) patients with CD at onset (n=100); ii) patients with potential CD (n=45); iii) patients with CD associated with other auto-immune diseases (n=17); and iv) patients with eosinophilic esophagitis (n=15). Among the patients with CD, 49 were also analyzed after six months on a gluten-free diet, while data were also available for 13 patients after one year on a gluten-free diet. No significant differences were found in the circulating levels of TRAIL between the patients with CD and the patients with either eosinophilic esophagitis or potential CD. Patients with CD associated with other auto-immune diseases showed significantly lower levels of TRAIL when compared with patients with CD alone. The gluten-free diet did not significantly modify the levels of circulating TRAIL at 6 or 12 months. Thus, although T1D and CD share common immunological features, the circulating levels of TRAIL show a significant difference between the two pathologies, and do not appear to be modulated in CD.

## Introduction

Celiac disease (CD) is an autoimmune-mediated enteropathy, characterized by gluten-triggered small bowel mucosal lesions in genetically susceptible individuals. The current diagnostic criteria for CD require intestinal mucosal villous atrophy and the presence of serum anti-transglutaminase type 2 (anti-TG2) antibodies, both of which disappear when the patient adopts a gluten-free diet (GFD) (1). Patients with CD have an increased risk of developing other autoimmune disorders, in particular, type 1 diabetes (T1D) (2). T1D and these pathologies are considered to be related by a common genetic background. All of these diseases are associated with organ-specific autoantibodies that can be detected prior to the development of clinical symptoms. In particular, the first association between CD and T1D was suggested in 1969, and the genetic risk factors associated with the two diseases include human leukocyte antigen (HLA) genes and non-HLA genes (3).

In this context, previous studies have suggested that the TNF superfamily member TNF-related apoptosis-inducing ligand (TRAIL), a mediator of the immune system with anti-cancer activity (4,5), also plays an important role in the control of autoimmune diseases (6), and in particular in T1D (7–9). As compared with other TNF family members, TRAIL has a complex biology since it interacts with four transmembrane receptors (TRAIL-R1, -R2, -R3 and -R4) (4) and one soluble receptor (osteoprotegerin) (10).

On these bases, the aim of the present study was to analyze the serum levels of TRAIL in patients with celiac disease, studying a pediatric retrospective cohort, including patients with overt CD prior to and following 6–12 months on a gluten-free diet, patients with potential CD, patients with CD associated with other autoimmunities, and patients with eosinophilic esophagitis.

## Materials and methods

### Study population

Sera of pediatric individuals followed at the Institute for Maternal and Child Health - IRCCS ‘Burlo Garofolo’ of Trieste, Italy were obtained from: i) patients with CD at onset (n=100) and from the same CD patients after six (n=49) and 12 (n=13) months of gluten-free diet; ii) patients with potential CD (n=45); iii) patients with CD associated with other auto-immune diseases (n=17); and iv) patients with eosinophilic esophagitis (n=15). Parents/caregivers of all patients provided informed consent to blood sample drawing and storage for research purposes, in accordance with the Declaration of Helsinki of 1975. Data reported in Table I include the age and gender of the patients and the diagnosis. The study was approved by the Independent Bioethics Committee of the Institute for Maternal and Child Health-IRCCS ‘Burlo Garofolo’ (Trieste, Italy).

### TRAIL ELISA assay

Serum levels of TRAIL were measured in duplicate with a Human TRAIL/TNFSF10 Quantikine ELISA kit (R&D Systems, Minneapolis, MN, USA) following the manufacturer’s instructions, as previously described (11,12). Selected samples were run in each ELISA plate as internal controls, confirming the reproducibility of determinations over time.

### Statistical analysis

Box plots were used to represent the distribution of values in different groups of patients. After verifying that TRAIL serum values did not distribute normally (skewness and kurtosis joint normality test), the non-parametric Mann-Whitney rank-sum test was applied to compare the TRAIL values among different populations. The Wilcoxon signed-rank test for paired samples was used to compare the levels of TRAIL at onset with those following 6 and 12 months on a gluten-free diet. Correlation coefficients were calculated using Spearman’s rank correlation coefficient rho. A P-value <0.05 was considered to indicate a statistically significant difference. All analyses were conducted using Stata/IC 11.2 software for Windows (Stata Corp LP, College Station, TX, USA).

## Results

### Determination of the levels of circulating TRAIL in patients with CD at onset

**Figure 1:**
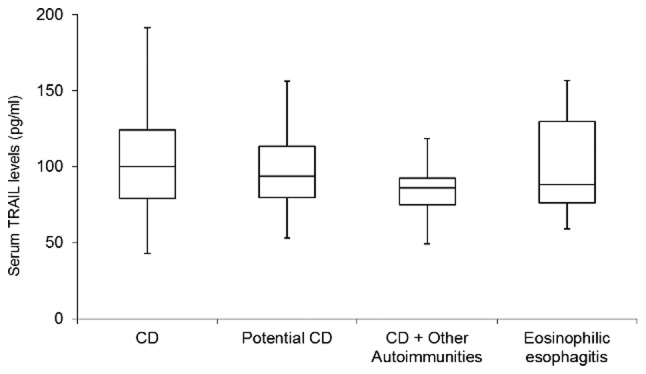
Levels of circulating TNF-related apoptosis-inducing ligand (TRAIL) in patients with different pathologies. CD, celiac disease.

As mentioned in Materials and methods, the cohort of individuals analyzed in the present study included: 100 patients with CD at onset, 45 patients with potential CD, 17 patients with CD associated with another autoimmunity, and 15 patients with eosinophilic esophagitis (Table I). No significant differences among the groups were observed in relation to the male to female ratio and no significant correlation was observed between serum TRAIL levels and the age of the patients. The circulating levels of TRAIL assessed in patients with CD were not significantly different when compared with those in patients with potential CD, nor when compared with those in patients with eosinophilic esophagitis (Table I, Fig. 1). Notably, the analysis of TRAIL levels between patients with CD with and without other concomitant autoimmune disorders (T1D, Hashimoto’s thyroiditis, Addison’s disease, vitiligo, autoimmune atrophic gastritis and psoriasis) revealed significantly lower levels in patients with other autoimmune disorders (P=0.047). This suggests that the concomitant presence of other autoimmune diseases impacts on the circulating levels of TRAIL in CD.

### Determination of the levels of circulating TRAIL in patients with CD at follow-up

**Figure 2:**
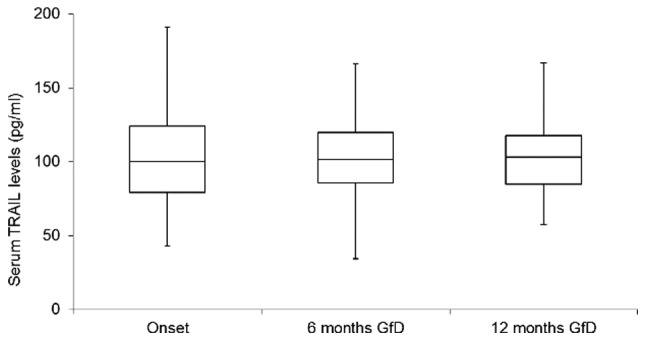
Levels of circulating TNF-related apoptosis-inducing ligand (TRAIL) in patients with celiac disease at onset and following 6 and 12 months of a gluten-free diet (GfD).

In the next group of experiments, in the cohort of CD patients, the serum levels of TRAIL were evaluated after 6 and 12 months on a gluten-free diet. TRAIL values at disease onset were not significantly different from the levels measured after six months on a gluten-free diet (n=49; P=0.773; Fig. 2). These data were confirmed also for a small subset of patients (n=13) for which data were also available after 1 year of a gluten-free diet (difference between onset and 12 months: P=0.279; difference between 6 and 12 months: P=0.553).

## Discussion

By analyzing a cohort of pediatric patients affected by CD, the current study has demonstrated that the circulating levels of TRAIL are not significantly different in CD patients with respect to those in patients with potential CD or eosinophilic esophagitis, while the circulating levels of TRAIL are significantly lower in patients with CD and concomitant autoimmune disorders when compared with those in CD patients with no such disorders. In addition, the levels of circulating TRAIL remained stable over a 6–12 month follow-up performed on a fraction of the same CD patients that continued to receive a gluten-free diet.

From these results, it may be inferred that the behavior of circulating TRAIL profoundly differs between T1D and CD. In fact, it has previous been demonstrated that circulating levels of TRAIL significantly decrease in T1D, with the lowest levels of TRAIL being documented in T1D patients with diabetic ketoacidosis (DKA) and depending on the severity of the disease (10). Moreover, TRAIL serum levels at T1D onset showed an inverse correlation with the insulin requirement at different time points up to 21 months of follow-up (10) and these data were in agreement with an independent study performed on T2D, which showed that circulating levels of TRAIL increased in patients with insulin therapy (13).

The present study suggests that the association between the circulating levels of TRAIL and the severity of the auto-immune reaction should be further explored, taking into account that CD patients with other concomitant auto-immune diseases showed significantly lower levels as compared with those in CD patients without concomitant auto-immune diseases. The lack of a correlation between the natural history and/or the response to therapy in CD, and the circulating levels of TRAIL in CD is suggested by the lack of differences observed between CD patients prior to and following a gluten-free diet. By contrast, the fact that TRAIL levels are significantly lower in T1D patients with DKA at onset and with a higher insulin requirement, points to a possible association between TRAIL and the metabolic stress underlying T1D (10). Thus, unlike with other cytokines, which appear to be modulated in CD (14–16), circulating levels of TRAIL do not appear to change, despite the ability of TRAIL to modulate the immune response in different experimental settings. It remains to be demonstrated whether TRAIL might be modulated at the gut level.

## Figures and Tables

**Table I:** Description of the sample of patients on which circulating levels of TRAIL were measured.

Pathologies	No. (%)	Age, years^a^	Male gender (%)	TRAIL, pg/ml^a^	P-value^b^
CD	100 (56.5)	6.3 (3.7–9.9)	35 (35)	100.0 (79.3–124.2)	Reference
Potential CD	45 (25.4)	6.6 (3.9–10.7)	20 (44)	93.6 (79.8–113.3)	0.315
CD + other autoimmunity	17 (9.6)	9.1 (4.1–16.5)	4 (24)	86.0 (75.1–92.4)	0.047
Eosinophilic esophagitis	15 (8.5)	12.8 (10.1–15.6)	12 (80)	88.1 (75.6–138.0)	0.684
Total	177 (100.0)	7.1 (3.9–11.0)	71 (40)	94.7 (78.9–119.9)	

TRAIL, TNF-related apoptosis-inducing ligand; CD, celiac disease.

a Median and interquartile range

b Mann-Whitney rank sum test for the comparison of the values of TRAIL between groups, P-value of comparison with the CD group.

## References

[1] Green PH, Cellier C (2007). Celiac disease. N Engl J Med.

[2] Scaramuzza AE, Mantegazza C, Bosetti A, Zuccotti GV (2013). Type 1 diabetes and celiac disease: The effects of gluten free diet on metabolic control. World J Diabetes.

[3] Hooft C, Roels H, Devos E (1969). Diabetes and coeliac disease. Lancet.

[4] Secchiero P, Zauli G (2008). TNF-related apoptosis-inducing ligand and the regulation of hematopoiesis. Curr Op Hematol.

[5] Secchiero P, Gonelli A, Celeghini C (2001). Activation of the nitric oxide synthase pathway represents a key component of tumor necrosis factor-related apoptosis-inducing ligand-mediated cytotoxicity on hematologic malignancies. Blood.

[6] Vinay DS, Kwon BS (2011). The tumour necrosis factor/TNF receptor superfamily: therapeutic targets in autoimmune diseases. Clin Exp Immunol.

[7] Di Bartolo BA, Chan J, Bennett MR, Cartland S, Bao S, Tuch BE, Kavurma MM (2011). TNF-related apoptosis-inducing ligand (TRAIL) protects against diabetes and atherosclerosis in ApoE^−/−^ mice. Diabetologia.

[8] Zauli G, Toffoli B, di Iasio MG, Celeghini C, Fabris B, Secchiero P (2010). Treatment with recombinant tumor necrosis factor-related apoptosis-inducing ligand alleviates the severity of streptozotocin-induced diabetes. Diabetes.

[9] Tornese G, Iafusco D, Monasta L (2014). The levels of circulating TRAIL at the onset of type 1 diabetes are markedly decreased in patients with ketoacidosis and with the highest insulin requirement. Acta Diabetol.

[10] Zauli G, Melloni E, Capitani S, Secchiero P (2009). Role of full-length osteoprotegerin in tumor cell biology. Cell Molecular Life Sci.

[11] Volpato S, Ferrucci L, Secchiero P (2011). Association of tumor necrosis factor-related apoptosis-inducing ligand with total and cardiovascular mortality in older adults. Atherosclerosis.

[12] Secchiero P, Corallini F, Ceconi C, Parrinello G, Volpato S, Ferrari R, Zauli G (2009). Potential prognostic significance of decreased serum levels of TRAIL after acute myocardial infarction. PloS One.

[13] Secchiero P, Corallini F, Beltrami AP (2010). An imbalanced OPG/TRAIL ratio is associated to severe acute myocardial infarction. Atherosclerosis.

[14] Xiang G, Zhang J, Ling Y, Zhao L (2013). Circulating level of TRAIL concentration is positively associated with endothelial function and increased by diabetic therapy in the newly diagnosed type 2 diabetic patients. Clin Endocrinol (Oxf).

[15] Lahdenperä AI, Hölttä V, Ruohtula T (2012). Up-regulation of small intestinal interleukin-17 immunity in untreated coeliac disease but not in potential coeliac disease or in type 1 diabetes. Clin Exp Immunol.

[16] Pagliari D, Cianci R, Frosali S, Landolfi R, Cammarota G, Newton EE, Pandolfi F (2013). The role of IL-15 in gastrointestinal diseases: A bridge between innate and adaptive immune response. Cytokine Growth Factor Rev.

